# Supplementary material for: Causal Relationship between Aging and Anorexia Nervosa: A White-Matter-Microstructure-Mediated Mendelian Randomization Analysis
Source: Biomedicines. 2024 Aug 16;12(8):1874. doi: 10.3390/biomedicines12081874 (PMC11351342; doi:10.3390/biomedicines12081874)
Supplement: Supplementary file 1 [file biomedicines-12-01874-s001.zip › Supplementary Figure/Figure S8/Leaveoneout plot of MR analysis for TL to ubm-b-1809.pdf]

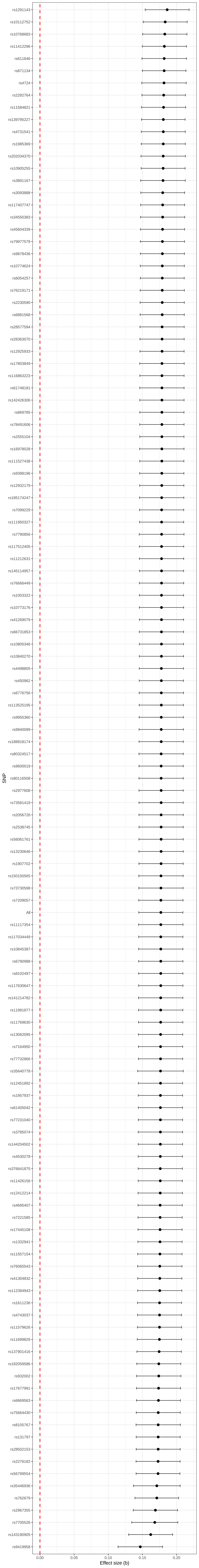
rs1291143
rs10112752
rs10768683
rs11412296
rs611646
rs871134
rs4724
rs2282764
rs11584821
rs139795227
rs4731541
rs1985369
rs202034370
rs10905255
rs3891167
rs3093888
rs117407747
rs34550383
rs45604339
rs79977579
rs9878436
rs10774624
rs6054257
rs76219171
rs2230590
rs6881568
rs28577594
rs28363070
rs12925933
rs17803849
rs116863223
rs61748181
rs142426306
rs869785
rs78491606
rs2555104
rs16978028
rs111527438
rs9398196
rs12932179
rs185174247
rs7099229
rs111950327
rs7790856
rs117512405
rs11212631
rs145114957
rs76666449
rs1003322
rs10773176
rs41269079
rs66731853
rs10805346
rs10840270
rs4498805
rs450962
rs6776756
rs113525195
rs9955360
rs9940099
rs188918174
rs80324517
rs9600019
rs80116508
rs2977608
rs73581419
rs2056726
rs2538745
rs56061761
rs13230646
rs1907702
rs150150565
rs73730598
rs7209057
All
rs11117354
rs117034449
rs10845387
rs6790988
rs8102497
rs117630647
rs141214782
rs11991877
rs11769630
rs13062095
rs7164950
rs77732866
rs35640778
rs12451892
rs1957937
rs61405042
rs77231040
rs3785074
rs144204502
rs4530278
rs376641875
rs11426156
rs12412214
rs4695407
rs7221585
rs17445108
rs1332941
rs11557154
rs76065543
rs41304832
rs112394943
rs1611236
rs4743037
rs11579626
rs11699829
rs137901416
rs182059586
rs932002
rs17677991
rs6669563
rs75664430
rs8105767
rs131797
rs28502153
rs2276182
rs56799554
rs35446936
rs762679
rs2967355
rs7705526
rs143190905
rs9419958
SNP
0.00
0.05
0.10
0.15
0.20
Effect size (b)
